# MicroRNA-17-5p inhibits thyroid cancer progression by suppressing Early growth response 2 (EGR2)

**DOI:** 10.1080/21655979.2021.1935137

**Published:** 2021-06-15

**Authors:** Xiang Geng, YangYang Sun, JinJin Fu, Liang Cao, Yuan Li

**Affiliations:** a Department of Thyroid and Breast Surgery, The Affiliated Changzhou No.2 People’s Hospital of Nanjing Medical University, Changzhou, China; b Department of Pathology, The Affiliated Changzhou No.2 People’s Hospital of Nanjing Medical University, Changzhou, China; c Department of Gastroenterology, The Affiliated Changzhou No.2 People’s Hospital of Nanjing Medical University, Changzhou, China; d Department of General Surgery, The Affiliated Changzhou No.2 People’s Hospital of Nanjing Medical University, Changzhou, China

**Keywords:** miR-17-5p, EGR2, thyroid cancer, progression, metastasis

## Abstract

miR-17-5p has been proved that play important roles in many kinds of tumors progression. This study aimed at explore the function and mechanism of miR-17-5p in thyroid cancer (TC). RT-qPCR was used to detect miR-17-5p and Early growth response 2 (EGR2) expression in TC tissues and cells. CCK8 and colony formation assay were used to analyze cell proliferation. Cell migration and cell invasion was detected by Wound-healing assay and Transwell assay. Detection of protein expression using Western blot analysis. Dual-Luciferase assay was used to analyze the relationship between miR-17-5p and EGR2. In vivo experiment was performed by establishing Xenograft animal model to observe the function of miR-17-5p. We found that miR-17-5p is significantly increased in thyroid cancer tissues and cells. miR-17-5p inhibition repressed cell proliferation, clonal formation, cell migration, and cell invasion in thyroid carcinoma. Moreover, miR-17-5p inhibition suppressed tumorigenesis in vivo. Dual-Luciferase assay and Western blotting assay further proved that miR-17-5p has a negative regulation to EGR2. EGR2 was decreased in TC tissues and cells. Overexpressed EGR2 inhibited the development of thyroid carcinoma both vivo and in vivo. EGR2 knockdown remarkably decreased the anti-cancer effect of miR-17-5p inhibition. miR-17-5p is a thyroid cancer oncomir by modulation of the EGR2.

## Graphical abstract

**Figure:**
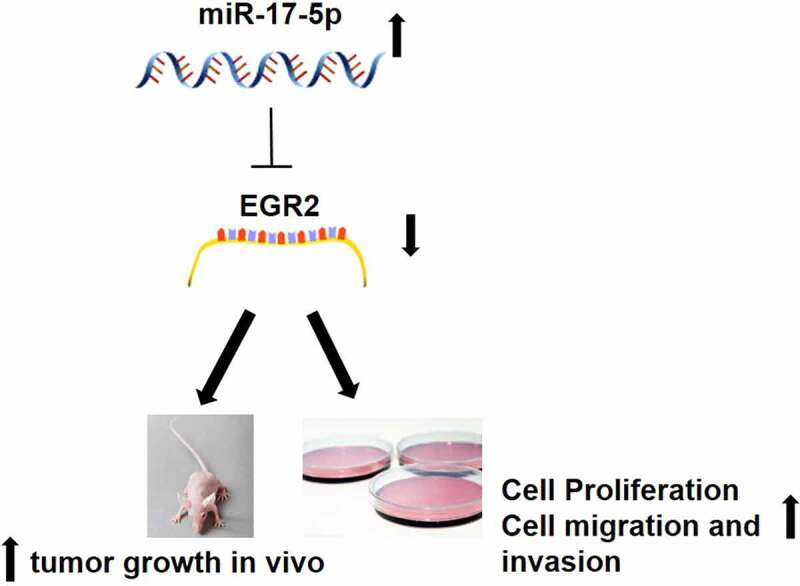

## Introduction

Thyroid cancer (TC) is one of the most popular malignancy of endocrine system globally. The incidence rate of thyroid cancer with a rapid worldwide increase over recent decades [1–3]. Although the treatment of thyroid cancer is improving, the overall 5-year survival rate of thyroid cancer patients is still not ideal [4–7]. Thus, it is very important to explore the pathogenesis and progression mechanism of TC, which may offer us new strategies for integrated treatment of TC patients.

miRNAs, a kind of small non-coding RNA molecules (length: approximately 22 nucleotides), has a variety of important regulatory roles in cells by causing mRNA degradation or translational suppression [8,9]. miRNAs inhibit the translation of target mRNA by directly cutting the target mRNA, or by fully or incompletely combining with the 3‘- UTR of target mRNA. The target mRNA further mediates a variety of biological behaviors, such as proliferation, differentiation, tumorigenesis [10,11]. Studies have shown that tumor tissues and normal tissues have a significantly different miRNA expression profile. Due to the role of target genes, some miRNAs can be carcinogenic or anticanceract genes, for example, mir-335-5p, mir-520A-3p and mir-718 play a significant role in the development of TC [12–14]. miR-17-5p has been shown to be overexpression in some forms of cancer such as gastric cancer and promote tumorigenesis and development [15–18]. In thyroid cancer, overexpression of miR-17-5p has been reported [19]. Knockdown of miR-17-5p suppressed cell proliferation and autophagy but promoted apoptosis in thyroid cancer cells. However, the underlying mechanism of miR‐17‐5p in TC has not been completely clarified [20].

Early growth response 2 (EGR2) has been shown to work as a zinc fnger transcription factor to regulate the development of nervous system [21]. Furthermore, EGR2 was found to be decreased in papillary thyroid carcinoma (PTC) tissues and cell lines and EGR2 could promote apoptosis in various cancers [22,23]. Furthermore, the interreaction between miRNAs and EGR2 was also reported. miR-20a promoted gastric cancer advance by regulating EGR2 [24]. miR-224-5p has also been reported that promote cell EMT in PTC by targeting EGR2 [22]. However, the revelence of miR-17-5p and EGR2 in TC is unknown.

This study aims to explore the expression of miR-17-5p and EGR2 level in TC, the molecular mechanism of miR-17-5p/EGR2 in promoting TC tumorigenesis both in vitro and in vivo, to lay a theoretical foundation for possible clinical application in the future.

## Materials and methods

### Ethical statement

This study was approved by The Affiliated Changzhou No.2 People’s Hospital of Nanjing Medical University ethics committee. All participants in this study signs a written informed consent.

### Clinical samples

Collecting 32 cases of human papillary thyroid cancer tissues and 32 paired non-tumor tissues from patients who received surgical therapy in the hospital. All samples were stored at −80°C.

### Cell culture

Cell culture of normal thyroid cell PTFE was using CM-H023 medium (Procell). Cell culture of thyroid carcinoma cell lines (TPC-1, B-CPAP, K1, and BHT101) were using RPMI-1640 medium (Life Technologies) supplemented with streptomycin-penicillin G and 10% FBS (Life Technologies). Cells were cultured in incubators at 37◦C with 5% CO2.

### RT-qPCR

Trizol (Invitrogen) extracted RNA, and then TaqMan microRNA reverse transcription Kit (Applied Biosystems) was used to transcribe RNA into cDNA. ABI step plus (Applied Biosystems) was used for PCR reaction. The relative miRNA expression was standardized by U6. After normalizing the housekeeping gene with 2 – Δ CT, the folding changes were measured relative to control group. The relative EGR2 expression was standardized by β-actin. The experiment was carried out at least three times. EGR2 forward:5ʹ-CGGTGACCATCTTCCCCAAT-3ʹ, and reverse: 5ʹ-GAGCGAAGCTACTCGGATACG-3ʹ. β-actin forward, 5′;-CTGGGACGACATGGAGAAAA-3′;; and reverse,

5′;-AAGGAAGGCTGGAAGAGTGC-3′;.

### Cell transfection

The lentiviral vectors of miR-17-5p or EGR2 were purchased from the GeneChem Company. The transfection was carried out according to the instruction of the producer using Lipo2000. The successfully transfected cells were collected for further experiments.

### CCK-8 analysis

CCK8 assay was carried out to analyze cell growth (Sigma). cells were added into 96-well plates at the cell concentration of 5 × 103 cells per well. Then detected the Optical Density (OD) at 450 nm.

### Colony formation analysis

Cells were inoculated in a 6-well culture plate with a concentration of 1000 cell/Wells. Cells cultured for 15 days and washing twice by PBS, 4% paraformaldehyde added to fix for 20 min, and staining with 1% crystal violet. Colonies were counted in five randomly selected areas under Olympus.

### Cell migration assay

Carrying out wound-healing test to analyze ability of cell migration. The cells were inoculated in a 6-well plates and cultured to 90% concentration. Making artificial wound using a 200 μl pipette and then changed medium to fresh medium. The cell debris was removed in free serum media. The migration ability was observed on Olympus at 24 hours.

### Transwell assay

Cell invasion was measured by Transwell method. The cells were seeded in Transwell chamber (pore size: 8 μm) according to the density of 1 × 10^4^ cells (100 μL) per well. 600 μl media containing 20% FBS were putted into the chamber below. The cells were placed in an incubator for 24 h. We take out the chamber, wipe off noninvasive cells with a cotton swabs. The invasive cells, which was attached to the sub-membrane surface, were mobilized with 90% ethanol for 10 min, rinse with 0.1% crystal violet for 5 min and PBS for 3 times and take photos under inverted microscope (Olympus). Randomly selected five fields for cell counting.

### Dual-luciferase reporter analysis

The 3ʹ UTR of human EGR2 or EGR2-Mut amplication was cloned into pGL3-promoter vector. These structures (1 g) were co-transfection with miR-17-5P inhibitor into TPC-1 cells. The luciferase activity was measured at least three times by Dual-luciferase activity analysis system (Promega) at 48 h after transfection.

### Western blotting analysis

RIPA peptide lysis buffer containing 1% protease inhibitor (Pierce) was used to lyse tissue samples or cells. BCA kit (Pierce) was choose to evaluate the protein concentration. Each sample (20 ug) electrophoresis using 10% sds-page, then we transferred protein into a nitrocellulose membrane (Santa cruz biotechnology). After sealing off in 5% skim milk, primary antibody was added into membrane at 4°C for overnight. After TBST washing for 3 times, secondary antibody was added into the membranes for 1 h, and then enhanced chemiluminescence (ECL) solution was used to protein develop. The protein expression was detected by ImageJ. β – actin was choose as a loading control.

### Immunohistochemistry

Paraffin section in dimethyl benzene dewaxing, then in turn antigen with grade alcohol after water recycling by the slice sample in 95 ◦C microwave boiling for 10 minutes in the EDTA (pH 6.0) biopsy samples at room temperature in 3% hydrogen peroxide in 30 minutes, and then use 20% goat serum closed 40 minutes biopsy samples with 20% goat anti EGR2 antibody in the serum culture (1:100) under the 4 ◦C overnight all slices at room temperature with HRP combined with protein secondary antibody incubation 60 minutes with 2 amino benzidine staining, slice with hematoxylin dyeing

### In vivo tumorigenesis assay

After purchasing male nude mice (BALB/c-nu, 4–5 weeks) and propagating under special pathogen-free (SPF) conditions, TPC-1 cells were transfection with miR-17-5P inhibitor or miR-NC and were subcutaneous injected into the left armpit of mice. Tumor volume and tumor weight were evaluated every 2 days, all mice were sacrificed at the end of the experiment, and tumor tissues were dissected and weighed.

### Statistical and analytical methods

The data are shown as mean ± SD. We did the statistical analysis using GraphPad Prism 5.0. A student’s t-test was used for the difference analysis between the two groups. One-way ANOVA test the difference among more than two groups. There was a significant difference when P < 0.05.

## Results

Thyroid cancer (TC) is one of the most popular malignancy of endocrine system globally. Understanding the pathogenesis and progression mechanism of TC, may offer us new strategies for integrated treatment of TC patients. miR-17-5p has been reported increased in TC. However, its roles and mechanisms in TC need further elucidation. In the present study, we conducted a series of in vitro and in vivo assays, aimed to explore the molecular mechanism of miR-17-5p/EGR2 in promoting TC tumorigenesis both in vitro and in vivo, to lay a theoretical foundation for possible clinical application in the future.

### miR-17-5p up-regulation promoted human TC malignancy

**Figure 1.**
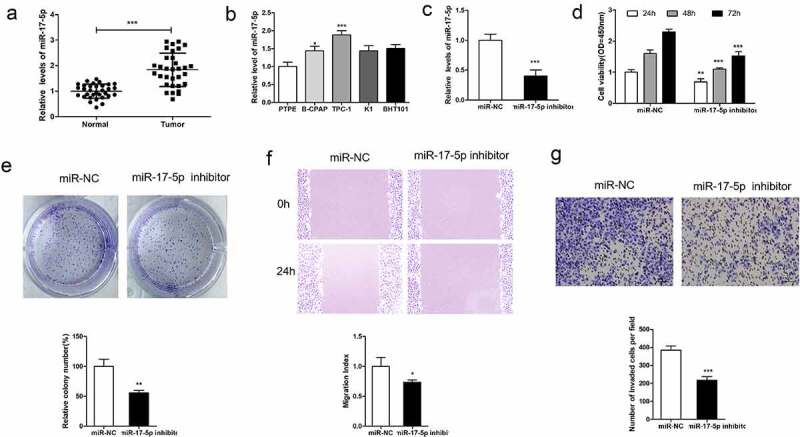
miR-17-5p overexpression promotes human thyroid cancer progression

We conducted RT-qPCR analysis on 32 pairs of thyroid tumor samples and non-tumor tissues to evaluate miR-17-5p expression level. As shown in Figure 1(a), miR-17-5p was remarkably up-regulated in thyroid carcinoma tissues in comparison with non-tumor tissues. miR-17-5p levels in normal thyroid cancer cells PTFE and four thyroid carcinoma cell lines (TPC-1, B-CPAP, K1, and BHT101) were also analyzed, as shown in Figure 1(b), miR-17-5p expressed differentially higher in the four kind of thyroid carcinoma cell lines than that in PTFE cells (Figure 1(b)). The function of miR-17-5p in thyroid carcinoma malignancy was then explored, TPC-1 cells, which have high miR-17-5p level, were transfection with miR-17-5p inhibitor or miR-NC. RT-qPCR analysis showed that miR-17-5p was down-regulated in cells transfection with miR-17-5p inhibitor in comparison with cells transfection with miR-NC (Figure 1(c)). CCK8 analysis, colony formation analysis, cell migration analysis, and cell invasion assays were carried out in TPC-1 cells, which transfection with miR-17-5p inhibitor or miR-NC. It turns out, miR-17-5p reduction significantly repressed cell growth and cell metastasis (Figure 1(d–g)). miR-17-5p might function as a tumor oncogene in thyroid carcinoma.

(a) RT-qPCR was used to analyze miR-17-5p level. (b) RT-qPCR analysis of miR-17-5p expression in four TC cell lines TPC-1, B-CPAP, K1, and BHT101 and one normal cell line PTPE. (c) TPC-1 cells were transfection with anti-miR-17-5p lentivirus (miR-17-5p inhibitor), RT-qPCR analysis of miR-17-5p level. (d) cell proliferation detected by CCK-8 assay. (e) Representative results of colony formation assay. (f) cell migration detection. (g) cell invasion detection. *P < 0.05, **P < 0.01, ***P < 0.001.

### miR-17-5p targeting EGR2 in thyroid carcinoma cells

**Figure 2.**
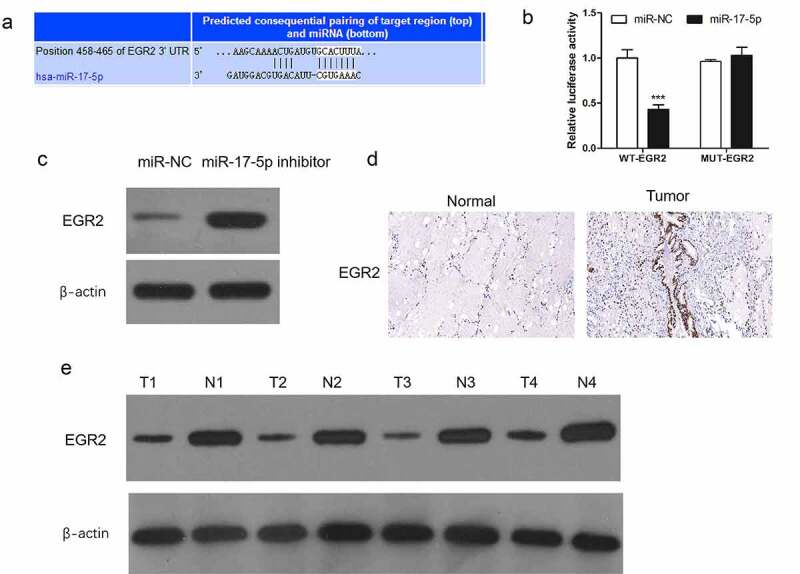
EGR2 is directly inhibited by miR-17-5p in thyroid cancer cells

The potential mechanisms of action of miR-17-5p in TC was searched. Targetscan was used to explore its target. miR-17-5p was predicted to bind with EGR2 directly in thyroid carcinoma (Figure 2(a)). To verify this, the luciferase reporter vector containing EGR2 3ʹ UTR or mutants was established. As shown in Figure 2(b), luciferase activities were strikingly weakened in TPC-1 cells which cotransfection with the WT-EGR2 and miR-17-5p, however, there was not observed in the cells co-transfection with the MUT-EGR2 and miR-17-5p. WB shown that miR-17-5p inhibitor transfection remarkably promoted EGR2 level (Figure 2(c)). Further, we measured EGR2 expression levels in thyroid carcinoma tissues by WB and immunohistochemistry. Our results showed that EGR2 levels were observely reduced in the TC tissues in comparison with paired normal tissues (Figure 2(d,e)).

(a) Identification of EGR2 as target of miR-17-5p by Targetscan. (b) Luciferase activity in TPC-1 cells. (c) detection of EGR2 level. β-actin was used as an internal control. (d) Immunohistochemistry analysis of EGR2 expression (100 ×). € detection of EGR2 level in TC tissues. ***P < 0.001.

### EGR2 overexpression inhibited thyroid cancer progression

**Figure 3.**
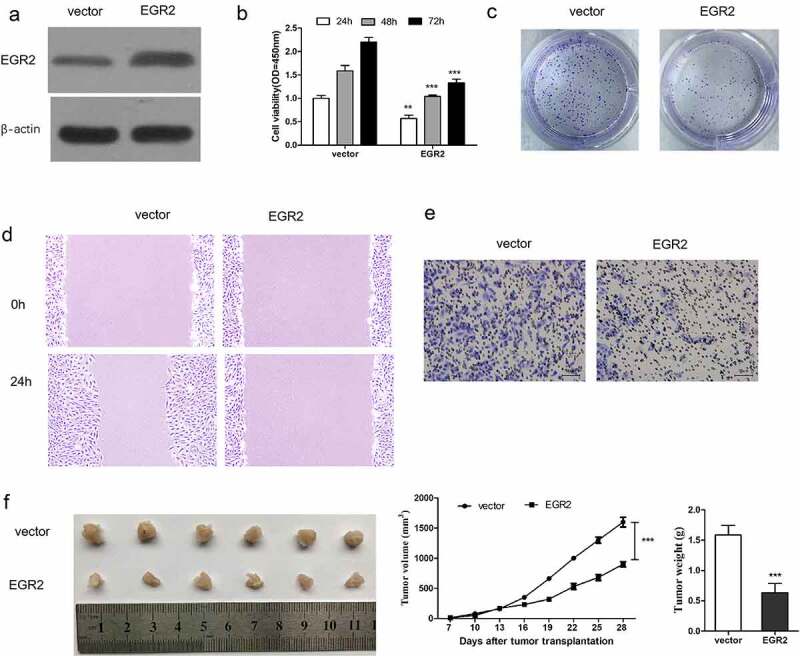
EGR2 overexpressed inhibited thyroid cancer progression

Due to EGR2 has been shown as a target gene of miR-17-5p, we next study the function of EGR2 in thyroid cancer progression. TPC-1 cells were transfected with a EGR2 overexpression vector to up-regulate EGR2 (Figure 3(a)). CCK8 analysis (Figure 3(b)), colony formation analysis (Figure 3(c)), cell migration analysis (Figure 3(d)) and Transwell analysis (Figure 3(e)) all confirmed that overexpression of EGR2 effectively inhibited cell proliferation and metastasis in vitro. Subcutaneous tumorigenesis in nude mice also demonstrated that EGR2 overexpression remarkably inhibited thyroid cancer progression in vivo (Figure 3(F)).

TPC-1 cells were transfection in EGR2 lentivirus (EGR2) and control (vctor). (a) WB analysis of EGR2 level. (b) detection of cell proliferation. (c) Representative photoes of colony formation assay. (d) detect of cell migration. (e) detection of cell invasion. (f) Images of xenograft tumors and Mean tumor weights are provided. ***P < 0.001.

### Downregulation of EGR2 reverses anti-tumor effects of miR-17-5p inhibitor in thyroid cancer cells

**Figure 4.**
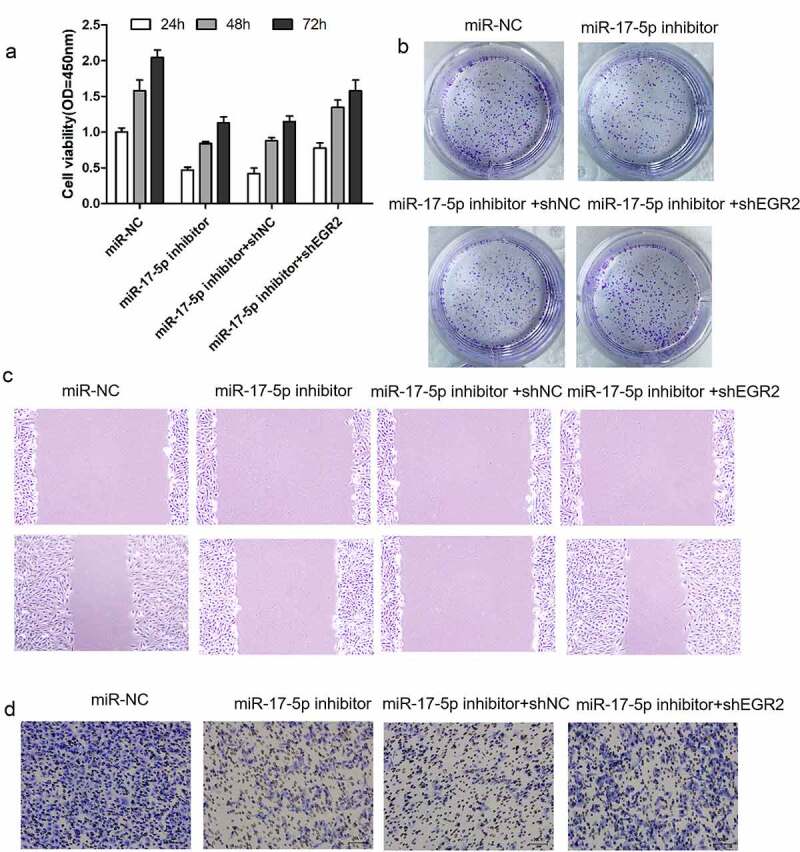
Downregulation of EGR2 reverses anti-tumor roles of miR-17-5p inhibition in TC cells

To explore the function relevance of miR-17-5p suppressing EGR2 in thyroid carcinoma, EGR2 was knockdown in TPC-1 cells. EGR2 inhibition significantly restored miR-17-5p-inhibitor-reduced cell growth, cell migration, and cell invasion in thyroid cancer (Figure 4(a–d)), indicating that miR-17-5p promotes thyroid carcinoma progression by suppressing EGR2.

(a) TPC-1 cells were cotransfected with shEGR2 and miR-17-5p inhibitor. A: detection of cell proliferation. (b) Representative results of colony formation assay. (c) detection of cell migration. (d) detection of cell invasion.

### Induction of tumor formation by miR-17-5p via inhibiting EGR2 in vivo

**Figure 5.**
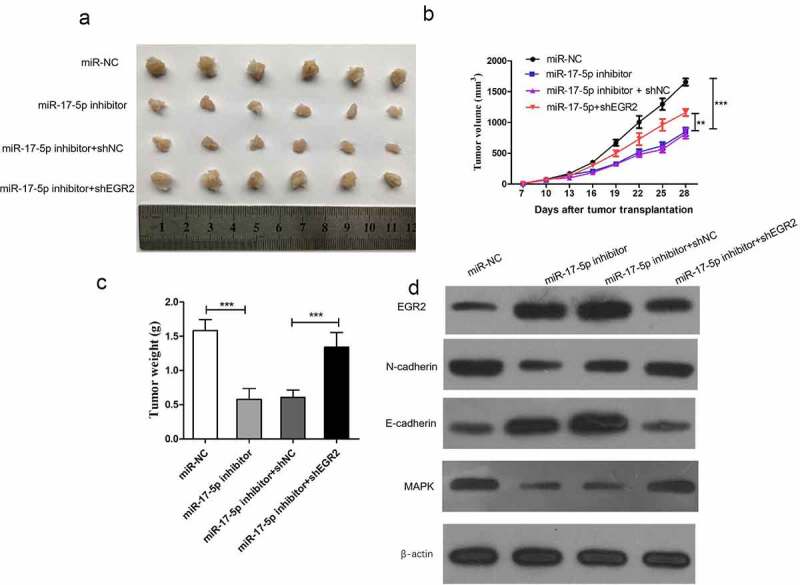
miR-17-5p induces TC tumorigenicity in vivo by suppressing EGR2

To investigate the function of miR-17-5p in vivo, we transfected TPC-1 cells with miR-17-5p inhibitor or miR-NC, the cells were then used to construct subcutaneous tumor model in nude mice. Tumor growth in miR-17-5p inhibition group was significantly slower than that in miR-NC group (Figure 5(a)). miR-17-5p downregulation group showed smaller tumor size and weight than control group (Figure 5(b,c)). We measured the levels of miR-17-5p and EGR2 in the tumor tissue. Compared with Mir NC group, miR-17-5p inhibitor group had up-regulated EGR2 expression (Figure 5(d)). Moreover, reduction of EGR2 could reverse anti-tumor effect of miR-17-5p inhibitor in vivo. Furthermore, the levels of E-cadherin and MAPK in tumor tissues from the miR-17-5p inhibition group were decreased in comparison with the control group (Figure 5(d)), indicating miR-17-5p promote thyroid cancer tumorigenicity by suppressing EGR2.

(a) Represented images of tumors. (b) Tumor volumes. (c) Tumor weights. (d) EGR2, E-cadherin, N-cadherin, and MAPK expression level in tumors. **P < 0.01, ***P < 0.001.

## Discussion

Here, we shown that miR-17-5p was apparently overexpression, while EGR2 was otherwise significantly down-regulation in thyroid cancer tissues as well as cell lines in comparison with the paired non-tumorl tissues and normal cells. Down‐regulation of miR-17-5p observably reduced cell growth, colony formation, cell migration and cell invasion of TC cells by inducing EGR2 expression. These results are expected to provide new strategies for thyroid carcinoma treatment.

A number of researches proved miRNAs are always making a significant contribution to the development of TC. For example, miR-613 could dramatically suppress cell growth and cell metastasis in PTC by modulating SphK2 expression [25]. miRNA‐338‐3p inhibited cell growth and metastasis while reduced apoptosis of thyroid carcinoma cells by mediating CCND1 [26]. miR-338-3p strikingly decreased TC malignancy by regulating AKT3 [27]. MiR-224-5p has been reported to direct binding with EGR2 to accelerate the progression of papillary thyroid carcinoma. Here, we revealed that miR-17-5p expression was notably increased in TC tissues and cell lines in comparison with paired non-tumor tissues and cell lines, which was consistent with previous studies [19,20]. Besides, miR-17-5p inhibitor reduced TC cell growth and cell metastasis, indicating that miR-17-5p might be a tumor oncogene in thyroid cancer.

Next, we explore its underlining mechanisms in human thyroid carcinoma advance. It has been reported that miR-17-5p could inhibited PTEN expression, therefore leading to suppression of the malignancy of thyroid cancer and inactivation of AKT/mTOR pathway, providing a novel avenue for treatment of thyroid cancer [20]. miR-17-5p targeting YES1 induced thyroid cancer progression [28]. In this study, Targetscan predicted that miR-17-5p might binding with EGR2 directly. Furthermore, Luciferase activity and Western blotting analysis further demonstrated that EGR2 was increased by miR-17-5p knockdown in TPC-1 cells. EGR2, a member of Egr family genes, encoded C2H2-type zinc-finger proteins [29]. EGR2 has been demonstrated play a crucial part in tumor development [30]. It is reported that EGR2 inhibited gastric cancer cell proliferation and cell invasion, which suggested that it might act as a antioncogene [31]. Overexpression of NFAT2 could significantly restrain hepatocellular carcinoma progression through increasing Egr2 expression [32]. Of note, downregulation of EGR2 expression were detected in PTC tissues and cells. EGR2 overexpression could attenuate the carcinogenic effects of miR-224-5p in PTC. In our study, we also found that EGR2 decreased in thyroid cancer tissues and cells. EGR2 overexpression could suppresses the malignancy of thyroid carcinoma through inhibition of tumor cell proliferation and migration. EGR2 knockdown significantly attenuate anti-cancer function of miR-17-5p. Turns out, miR-17-5p directly targeting EGR2 in TPC-1 cells. Given that EGR2 has a key role in inhibiting human tumors malignancy, targeting EGR2 may be an effective new therapy strategies for the prevention of tumor progression. Of note, in this study, miR-17-5p was illustrated to negatively modulate EGR2 in TPC-1 cells, further confirming a tumor oncogene role of miR-17-5p.

## Conclusion

In summary, our study demonstrates that the expression of miR-17-5p is up-regulation in TC tissues and cells. Inhibition of miR-17-5p reduces cell growth and metastasis via inhibiting EGR2 in TPC-1 cells. Furthermore, our results indicated EGR2 is the target gene of miR-17-5p in TC, which may supply a promising treatment target for thyroid cancer.

## Highlights

1. miR-17-5p is significantly increased in thyroid cancer tissues and cells.

2. mR-17-5p promotes thyroid cancer tumorigenesis both in vitro and in vivo.

3. miR-17-5p is a thyroid cancer oncomir by modulation of the EGR2.

## References

[1] Chen W, Zheng R, Baade PD, et al. Cancer statistics in China, 2015. CA Cancer J Clin. 2016;66(2):115–132. 26808342 10.3322/caac.21338

[2] Ruan X, Shi X, Dong Q, et al. Antitumor effects of anlotinib in thyroid cancer. Endocr Relat Cancer. 2019;26(1):153–164. 30139768 10.1530/ERC-17-0558 PMC6215907

[3] Liu Y, Zhang H, Wang H, et al. Long non-coding RNA DUXAP8 promotes the cell proliferation, migration, and invasion of papillary thyroid carcinoma via miR-223-3p mediated regulation of CXCR4. Bioengineered. 2021;12(1):496–506. 10.1080/21655979.2021.1882134 PMC8291844 33522355

[4] McFarland DC, Misiukiewicz KJ. Sorafenib in radioactive iodine-refractory well-differentiated metastatic thyroid cancer. Onco Targets Ther. 2014;7:1291–1299. 25053887 10.2147/OTT.S49430 PMC4105272

[5] Smallridge RC, Marlow LA, Copland JA. Anaplastic thyroid cancer: molecular pathogenesis and emerging therapies. Endocr Relat Cancer. 2009;16(1):17–44. 18987168 10.1677/ERC-08-0154 PMC2829440

[6] Pemayun TG. Current diagnosis and management of thyroid nodules. Acta Med Indones. 2016;48(3):247–257. 27840362

[7] Tuttle RM. Controversial issues in thyroid cancer management. J Nucl Med. 2018;59(8):1187–1194. 29653980 10.2967/jnumed.117.192559 PMC6071505

[8] Sun Z, Shi K, Yang S, et al. Effect of exosomal miRNA on cancer biology and clinical applications. Mol Cancer. 2018;17(1):147. 30309355 10.1186/s12943-018-0897-7 PMC6182840

[9] Lin S, Gregory RI. MicroRNA biogenesis pathways in cancer. Nat Rev Cancer. 2015;15(6):321–333. 25998712 10.1038/nrc3932 PMC4859809

[10] Gambari R, Brognara E, Spandidos DA, et al. Targeting oncomiRNAs and mimicking tumor suppressor miRNAs: Νew trends in the development of miRNA therapeutic strategies in oncology (Review). Int J Oncol. 2016;49(1):5–32. 27175518 10.3892/ijo.2016.3503 PMC4902075

[11] Di Leva G, Garofalo M, Croce CM. MicroRNAs in cancer. Annu Rev Pathol. 2014;9:287–314. 24079833 10.1146/annurev-pathol-012513-104715 PMC4009396

[12] Bi CL, Zhang YQ, Li B, et al. MicroRNA‐520a‐3p suppresses epithelial‐mesenchymal transition, invasion, and migration of papillary thyroid carcinoma cells via the JAK1‐mediated JAK/STAT signaling pathway. J Cell Physiol. 2018;4:4054‐4067. 10.1002/jcp.27199 30206929

[13] Wang X, Qi M. miR‐718 is involved in malignancy of papillary thyroid cancer through repression of PDPK1. Pathol Res Pract. 2018;214:1787‐1793. 10.1016/j.prp.2018.08.022 30166214

[14] Luo LI, Xia LI, Zha B, et al. miR‐335‐5p targeting ICAM‐1 inhibits invasion and metastasis of thyroid cancer cells. Biomed Pharmacother. 2018;106:983‐990. 10.1016/j.biopha.2018.07.046 30119270

[15] Song J, Liu Y, Wang T, et al. MiR-17-5p promotes cellular proliferation and invasiveness by targeting RUNX3 in gastric cancer. Biomed Pharmacother. 2020;128:110246. 32447210 10.1016/j.biopha.2020.110246

[16] Xu J, Meng Q, Li X, et al. Long noncoding RNA MIR17HG promotes colorectal cancer progression via miR-17-5p. Cancer Res. 2019;79(19):4882–4895. 31409641 10.1158/0008-5472.CAN-18-3880

[17] Cai N, Hu L, Xie Y, et al. MiR-17-5p promotes cervical cancer cell proliferation and metastasis by targeting transforming growth factor-β receptor 2. Eur Rev Med Pharmacol Sci. 2018;22(7):1899–1906. 29687841 10.26355/eurrev_201804_14712

[18] Zhao J, Xiao A, Liu C, et al. The HIF-1A/miR-17-5p/PDCD4 axis contributes to the tumor growth and metastasis of gastric cancer. Signal Transduct Target Ther. 2020;5(1):46. 32296039 10.1038/s41392-020-0132-z PMC7145839

[19] Takakura S, Mitsutake N, Nakashima M, et al. Oncogenic role of miR-17-92 cluster in anaplastic thyroid cancer cells. Cancer Sci. 2008;99(6):1147–1154. 18429962 10.1111/j.1349-7006.2008.00800.x PMC11160010

[20] Shi YP, Liu GL, Li S, et al. miR-17-5p knockdown inhibits proliferation, autophagy and promotes apoptosis in thyroid cancer via targeting PTEN. Neoplasma. 2020 Mar;67(2):249–258. 31973533 10.4149/neo_2019_190110N29

[21] Topilko P, Schneider-Maunoury S, Levi G, et al. Krox-20 controls myelination in the peripheral nervous system. Nature. 1994;371:796–799. 7935840 10.1038/371796a0

[22] Zang CS, Huang HT, Qiu J, et al. MiR-224-5p targets EGR2 to promote the development of papillary thyroid carcinoma. Eur Rev Med Pharmacol Sci. 2020;24(9):4890–4900. 32432752 10.26355/eurrev_202005_21178

[23] Unoki M, Nakamura Y. EGR2 induces apoptosis in various cancer cell lines by direct transactivation of BNIP3L and BAK. Oncogene. 2003;22:2172–2185. 12687019 10.1038/sj.onc.1206222

[24] Wei L, Ran F. MicroRNA-20a promotes proliferation and invasion by directly targeting early growth response 2 in non-small cell lung carcinoma. Oncol Lett. 2018;15(1):271–277. 29375712 10.3892/ol.2017.7299 PMC5766075

[25] Qiu W, Yang Z, Fan Y, et al. MicroRNA-613 inhibits cell growth, migration and invasion of papillary thyroid carcinoma by regulating SphK2. Oncotarget. 2016;7(26):39907–39915. 27223438 10.18632/oncotarget.9530 PMC5129980

[26] Guo F, Fu Q, Wang Y, et al. Long non-coding RNA NR2F1-AS1 promoted proliferation and migration yet suppressed apoptosis of thyroid cancer cells through regulating miRNA-338-3p/CCND1 axis. J Cell Mol Med. 2019;23(9):5907–5919. 31304680 10.1111/jcmm.14386 PMC6714216

[27] Sui GQ, Fei D, Guo F, et al. MicroRNA-338-3p inhibits thyroid cancer progression through targeting AKT3. Am J Cancer Res. 2017;7(5):1177–1187. 28560065 PMC5446482

[28] Liu L, Yang J, Zhu X, et al. Long noncoding RNA H19 competitively binds miR-17-5p to regulate YES1 expression in thyroid cancer. FEBS J. 2016;283(12):2326–2339. 27093644 10.1111/febs.13741

[29] Dzialo-Hatton R, Milbrandt J, Hockett RD Jr, et al. Differential expression of Fas ligand in Th1 and Th2 cells is regulated by early growth response gene and NF-AT family members. J Immunol. 2001;166(7):4534–4542. 11254710 10.4049/jimmunol.166.7.4534

[30] Unoki M, Nakamura Y. Growth-suppressive effects of BPOZ and EGR2, two genes involved in the PTEN signaling pathway. Oncogene. 2001;20(33):4457–4465. 11494141 10.1038/sj.onc.1204608

[31] Chen P, Zhao H, Huang J, et al. MicroRNA-17-5p promotes gastric cancer proliferation, migration and invasion by directly targeting early growth response 2. Am J Cancer Res. 2016;6(9):2010–2020. 27725906 PMC5043110

[32] Wang J, Zhang Y, Liu L, et al. NFAT2 overexpression suppresses the malignancy of hepatocellular carcinoma through inducing Egr2 expression. BMC Cancer. 2020;20(1):966. 33023539 10.1186/s12885-020-07474-0 PMC7542386

